# Synthetic Approaches for Nucleic Acid Delivery: Choosing the Right Carriers

**DOI:** 10.3390/life9030059

**Published:** 2019-07-09

**Authors:** Rong Ni, Ruilu Feng, Ying Chau

**Affiliations:** 1 Department of Chemical and Biological Engineering, the Hong Kong University of Science and Technology, Clear Water Bay, Kowloon, Hong Kong 999077, China; 2 Institute for Advanced Study, the Hong Kong University of Science and Technology, Clear Water Bay, Kowloon, Hong Kong 999077, China

**Keywords:** synthetic approach, nucleic acid delivery, intracellular barriers, endosomal escape, nuclear trafficking, synthetic carrier, artificial virus

## Abstract

The discovery of the genetic roots of various human diseases has motivated the exploration of different exogenous nucleic acids as therapeutic agents to treat these genetic disorders (inherited or acquired). However, the physicochemical properties of nucleic acids render them liable to degradation and also restrict their cellular entrance and gene translation/inhibition at the correct cellular location. Therefore, gene condensation/protection and guided intracellular trafficking are necessary for exogenous nucleic acids to function inside cells. Diversified cationic formulation materials, including natural and synthetic lipids, polymers, and proteins/peptides, have been developed to facilitate the intracellular transportation of exogenous nucleic acids. The chemical properties of different formulation materials determine their special features for nucleic acid delivery, so understanding the property–function correlation of the formulation materials will inspire the development of next-generation gene delivery carriers. Therefore, in this review, we focus on the chemical properties of different types of formulation materials and discuss how these formulation materials function as protectors and cellular pathfinders for nucleic acids, bringing them to their destination by overcoming different cellular barriers.

## 1. Introduction

The direct association between genetic mutations and various human diseases (such as cancer, HIV, and Alzheimer’s disease) has driven the development of efficient strategies for therapeutic purposes. Exogenous nucleic acids, including DNA and RNA, have been explored as therapeutic agents to treat these genetic disorders. They can either supplement deficient functional proteins through gene augmentation or suppress malfunctioning proteins through gene inhibition. Recent advances in gene therapy have been demonstrated in cancer treatment through (1) chimeric antigen receptor (CAR)-T immunotherapy, which reprograms T-cells with chimeric antigen receptor (CAR)-encoded long plasmid DNA (pDNA); (2) DNA vaccines, which use pDNA-encoded antigens to induce immune responses and activate cytotoxic T cells; (3) CRISPR-Cas9 (Clustered Regularly Interspaced Short Palindromic Repeats) genome editing, which engineers immune cells or corrects cancer-causing mutations with the assistance of enzymes and guide RNA; and (4) RNA-interference, which inhibits the expression of carcinogenic genes or apoptotic-resistant genes with short small interfering RNA (siRNA) [1,2,3,4]. The challenges for gene therapy include the following: (1) Nucleic acids easily degrade through chemical or enzymatic digestion, and (2) the hydrophilic nature and negatively charged chemical properties of nucleic acids restrict their passage through cell membranes. Therefore, the condensation and protection of nucleic acids with appropriate vehicles are required for successful gene therapy.

Viruses, natural pathogens, have evolved to protect and deliver the viral genome into host cells for infection. This inherited property has driven the extensive exploration of viruses as delivery vectors for gene therapy. Adeno-associated virus (AAV) was the first FDA-approved virus-based gene delivery vector for the treatment of a rare inherited retinal disease [5]. However, the potential risks of viruses, including insertional mutagenesis, high immunogenicity, and limited gene capacity [5], drive the development of safe gene delivery vectors. Biocompatible materials, including natural and synthetic lipids, polymers, and proteins/peptides [6,7,8,9,10,11], have been extensively developed for this purpose. Cationic formulation materials efficiently condense the negatively charged nucleic acids in the formulation material matrix through electrostatic interaction, which allows gene protection from enzymatic digestion. On the other hand, these formulation materials also function as cellular pathfinders for nucleic acids and bring them to the correct cellular location [12,13]. For exogenous nucleic acids, there are multiple cellular barriers on the way to their destinations [14], including (1) the negatively charged cell membrane, which prevents the entrance of negatively charged hydrophilic nucleic acids; (2) the endosome/lysosome vesicles, which have the potential to degrade the trapped nucleic acids [15]; (3) stable nucleic acid carriers, which prevent the function of nucleic acids from binding with cellular machineries; (4) viscous cytoplasm, which hampers the movement of DNA towards the nucleus [16]; (5) the nuclear envelope, which stops the entry of DNA into the nucleus [17]. Overcoming these different barriers requires the appropriate and well-orchestrated interaction between nucleic acid delivery carriers and the cellular compartments.

Therefore, understanding the properties of formulation materials and their correlated nucleic acid delivery functions is critical for us to develop applicable vehicles for specific types of exogenous nucleic acids. The recent development of different types of formulation materials and their biomedical applications in gene therapy field have already been systematically reviewed [13,14,15,16,17,18,19,20,21,22,23,24,25,26,27,28,29]. However, a parallel comparison of different formulation materials together with an analysis of the correlation of their material property–nucleic acid delivery function has not been conducted yet. Therefore, in this review, we choose well-studied formulation materials as representatives to discuss how the inherited properties of different formulation materials influence the delivery of nucleic acids. We attempt to answer the following questions: (1) How do different types of formulation materials achieve nucleic acid condensation and protection? (2) How do different formulation materials facilitate the cellular internalization of nucleic acids? (3) What is the mechanism used by each type of formulation material to achieve endosomal escape? (4) What are the strategies leading to nucleic acid release from the carriers? (5) How do different formulation materials carry nucleic acids toward the nucleus? (6) What are the strategies used for the active entry of DNA into nucleus? Finally, we summarize the properties of different types of formulation materials and the prospects for the development of next-generation gene delivery vehicles.

## 2. Natural and Synthetic Formulation Materials-Induced Nucleic Acid Condensation and Protection

**Figure 1:**
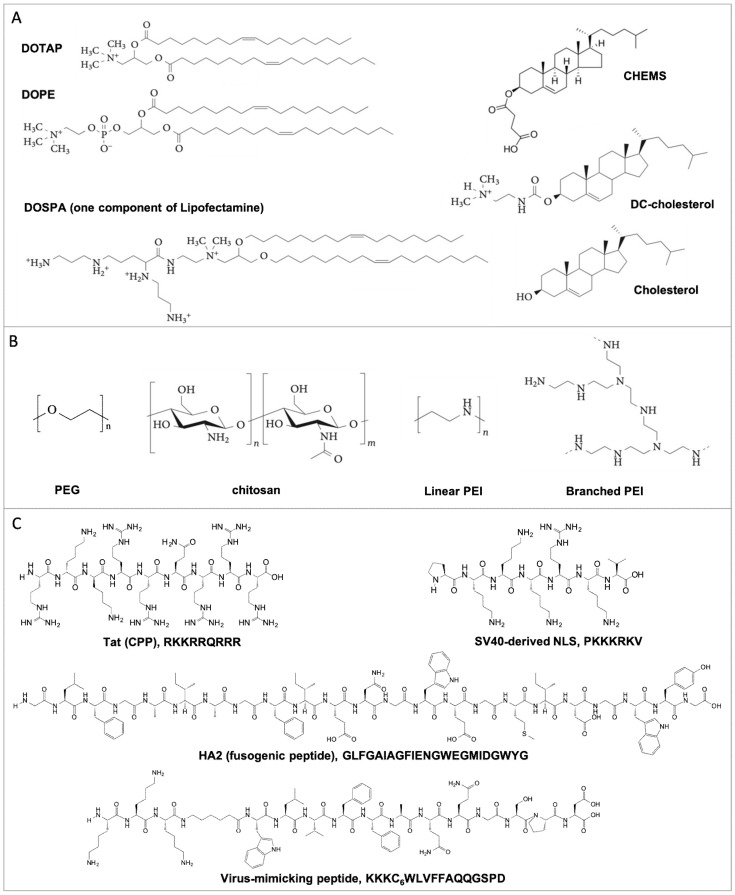
Chemical structure of the representative formulation materials, including (**A**) lipids, (**B**) polymers and (**C**) peptides. **Abbreviations:** DOTAP: 1,2-dioleoyl-3trimethylammonium-propane; DOPE: dioleoylphosphatidyl ethanolamine; CHEMS: cholesteryl hemisuccinate; DC-cholesterol: 3β-[N-(dimethylaminoethane)carbamoyl] cholesterol; DOSPA: 2,3-dioleyloxy-N-[2(sperminecarboxamido)ethyl]-N,N-dimethyl-1-propanaminium trifluoroacetate; PEG: polyethylene glycol; PEI: polyethylenimine; CPP: cell penetrating peptide; SV40: simian virus 40; NLS: nuclear localization signal; HA2: hemagglutinin 2.

Nucleic acids, including long plasmid DNA (pDNA) and messenger RNA (mRNA), and short oligodeoxynucleotide (ODN), microRNA (miRNA), small interfering RNA (siRNA), and small guide RNA (sgRNA), share similar chemical components (e.g., sugar, base, and phosphate groups), which define their hydrophilic and negatively charged nature. Therefore, the condensation of nucleic acids with cationic formulation materials is advantageous to (1) condense macromolecular nucleic acids into nano-sized particles; and (2) protect nucleic acids from degradation. In the following section, we focus on the general properties of different formulation materials (including lipids, polymers, and peptides/proteins), their condensation propensities, and the physical properties of the resulting nucleic acid complexes. The chemical structure of the representative formulation materials is summarized in Figure 1. The complexes with different structures, formulations, and physical properties display different mechanisms of transfection through different cellular internalization pathways, different endosomal escaping mechanisms, and different nuclear entry principles, which will be discussed in Section 3, Section 4, Section 5, Section 6 and Section 7.

### 2.1. Lipid-Based Lipoplexes

**Figure 2:**
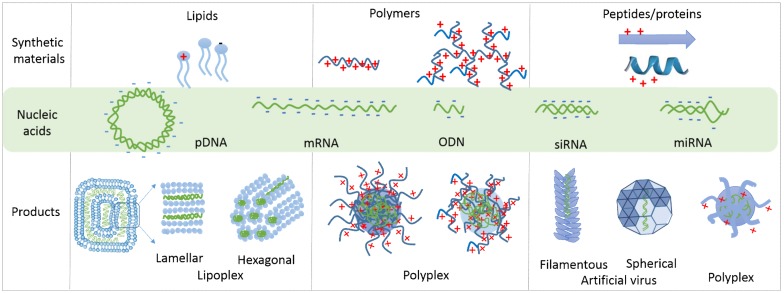
Condensation of nucleic acids with different formulation materials. These formulation materials include lipids, linear and branched polymers, and peptides/proteins with either natural or synthetic sources. The nucleic acids include pDNA (plasmid DNA), mRNA (messenger RNA), ODN (oligodeoxynucleotide), siRNA (small interfering RNA), and miRNA (microRNA). The complexing products of lipids/nucleic acids lead to lipoplexes with representative lamellar or hexagonal structures. The polymers and nucleic acids complex into polyplexes with polymer chains tangling together without any ordered internal structure. Peptides/proteins interact with nucleic acids to form either disordered polyplexes or ordered artificial viruses with filamentous or spherical morphologies, depending on the primary structure of the peptide/protein.

Lipids are amphiphilic molecules, consisting of hydrophobic tails and hydrophilic heads. According to the charge(s) carried by their head groups, lipids are classified into (1) cationic lipids (DOTAP), (2) neutral lipids (cholesterol, DOPE), (3) anionic lipids (CHEMS), and (4) pH-sensitive lipids (DOSPA) (Figure 1) [30,31]. The mixing of negatively charged nucleic acids with positively charged liposomes leads to the spontaneous formation of lipoplexes [32]. Structural characterization shows a defined lamellar structure (L_a_^c^), inverted hexagonal structure, and honeycomb structure (H_I_^c^) as thethree main structures of lipoplexes (Figure 2) [33,34,35]. The formation of these structures is driven by the topologies and chemical properties of the lipids, as well as their lipid to nucleic acid ratio, which has been reviewed elsewhere [36,37,38]. Their lipid properties and lipid:DNA ratio also influence the stability and cytotoxicity of the lipoplexes, as well as their size and surface charge [39,40]. Specifically, cationic lipids, the main components of the lipoplexes, have the potential to induce cytotoxicity and particle instability [41,42,43]. Therefore, incorporation of polyethylene glycol (PEG)-lipid conjugate (Figure 1) [44] and the addition of neutral lipids in lipoplex formulations have been frequently employed to decrease cytotoxicity and enhance the stability of the lipoplexes [45,46]. Another strategy to address cytotoxicity issue is to develop pH-sensitive ionizable lipids, which carry no charges in a neutral environment and are cationic at acidic pH. The examples include ionizable DOSPA in lipofectamine (Figure 1) [47] and DLin-MC3-DMA ((6Z,9Z,28Z,31Z)-heptatriaconta-6,9,28,31-tetraen-19-yl-4-(dimethylamino) butanoate) in lipid nanoparticles. The later lipid nanoparticles have already been approved by the FDA with the purpose of siRNA delivery for the treatment of polyneuropathy caused by transthyretin (TTR) amyloidosis [48]. Therefore, the balance of permanently charged, neutral, and ionizable lipid populations is critical to develop biocompatible and stable lipoplexes.

### 2.2. Polymer-Based Polyplexes

Different from small molecular lipids, polymers of high molecular weight efficiently condense nucleic acids into stable polyplexes with varied sizes and shapes (Figure 2) [28,49,50]. The most-studied polymers include cationic polymers, such as polyethyleneimine (PEI) (Figure 1) [51], which has been developed as one of the “gold standard” transfection reagents for gene delivery [10,52,53]. Similar to cationic lipoplexes, the positively charged polyplexes tend to induce cytotoxicity. Therefore, conjugation of the neutral moieties with PEI molecules, such as PEG- [54] and cyclodextrin (CD)- [55], has been performed to lower the surface charge and solve the cytotoxicity issue. Polymers of low molecular weight show less of a cytotoxicity problem, but these polymers also compromise the stability of the resulting polyplexes [56,57]. Therefore, to make polyplexes of higher stability and lower cytotoxicity, researchers usually conjugate hydrophobic moieties with polymers. For example, Chiper et al. chemically linked salicylamide with PEI (1.8 kDa) and the resulting PEI–salicylamide conjugate condensed DNA and RNA efficiently [58]. Zhao et al. conjugated stearic acid with PEI (2.0 kDa), which led to stable polyplexes with mRNA [59]. In addition, the polymer-to-nucleic acid charge ratio also influences the stability of the polyplexes. Wu et al. demonstrated that an N/P ratio higher than 6 was required for the formation of stable polyplexes [60]. The polymer topology determines the condensation mechanism [60]. Wu et al. discovered that linear PEI aligned along the DNA backbone to neutralize DNA, leading to DNA condensation, which easily dissociated through anionic dextran exchange [60], while branched PEI tangled with DNA, and the resulting polyplexes had difficulty releasing DNA through polyanionic exchange with dextran.

In addition, biocompatible and biodegradable polymers, such as chitosan (Figure 1) and PLGA (poly(lactic-co-glycolic acid), have been used for nucleic acid delivery. Chitosan is a nature-derived cationic polysaccharide, and condenses nucleic acids efficiently through electrostatic interaction. The ratio of the acetylated to deacetylated amino groups define the water solubility and charge density of chitosan. Highly deacetylated chitosan displayed great siRNA encapsulation and delivery efficiency [61]. In addition, the molecular weight of chitosan also influences the stability of the polyplexes. Polyplexes formed from chitosan of a high molecular weight had very high stability, which limited nucleic acid release inside cells, while chitosan of a low molecular weight (Mw ~16 kDa) allowed efficient nucleic acid release [62]. PLGA is an FDA-approved drug delivery formulation material. However, the neutral property of PLGA results in the encapsulation of nucleic acids in the PLGA matrix, mainly through water/oil/water double emulsion or nanoprecipitation [63]. This preparation process has two potential drawbacks, including that (1) The used organic solvent may degrade nucleic acids and (2) the encapsulation efficiency of nucleic acids is low. Therefore, the addition of cationic components, such as PEI, protamine, and DOTAP [63,64], significantly enhanced the encapsulation efficiency of nucleic acids, which also avoided the degradation of nucleic acids by organic solvents.

### 2.3. Peptide-Based Polyplexes

Peptides and proteins, a large category of degradable biopolymers, have been extensively explored for their role in nucleic acid delivery. These include cell-penetrating peptides (CPP) and nuclear localization signals (NLSs), which are rich in cationic amino acids (Figure 1). However, both groups of peptides are too short to form stable polyplexes with nucleic acids [65], so the chemical conjugation of CPP and NLS with other hydrophobic moieties has been applied. For example, the conjugation of a lipid alkyl chain with CPP/NLS [66] or a hydrophobic fusion sequence MPG (GALFLGFLGAAGSTMGAWSQPKKKRKV) (derived from HIV protein gp41) with a hydrophilic NLS peptide (KKKRKV derived from the SV40 virus) [67,68] enhanced the stability of the resulting polyplexes. Cross-linking of the short peptides through disulfide bonds is another strategy to develop stable polyplexes. For example, Zhang et al. modified the termini of the peptide with cysteine residues, which formed stable polyplexes through disulfide bonds [69]. These hydrophilic CPP/NLS peptides interact with nucleic acids mainly through electrostatic interaction, leading to polyplex-like nanoparticles without an ordered internal structure (Figure 2). However, some amphipathic peptide strands, such as MPG, condense nucleic acids through both electrostatic interactions (peptide-nucleic acid) and hydrogen-bonding/hydrophobic interactions (peptide-peptide), and the formation of peptide secondary structures contributes to the overall stability of the polyplexes [67].

Poly-L-lysine (PLL) is a frequently studied cationic polypeptide, which shows strong interaction and encapsulation capability with nucleic acids. Modification of PLL with hydrophobic polymer block enhanced the stability of polyplexes and prolonged nucleic acid protection against nucleases [70,71]. Protamine is an arginine-rich protein and has also been extensively exploited to condense nucleic acids [72]. Huang et al. used protamine to condense sperm DNA into compact polyplexes. Many studies also reported the combination of lipids or polymers with protamine to increase the stability of the resulted polyplexes [26,72].

### 2.4. Nature-Inspired Artificial Viruses

Inspired by viruses [73], virus-like particles (VLPs) of a confined nano-sized structure have been constructed for nucleic acid delivery by assembling the virus capsid proteins with cargo nucleic acids [74,75]. Previous studies reported different VLPs for mRNA [76] and pDNA delivery [77]. However, VLP production is usually required to transfect both the virus coat protein-expression plasmid and the cargo plasmid into the same host cell or bacteria [77,78,79,80]. The in situ expressed virus coat proteins assemble into VLPs, with the cargo pDNA being encapsulated inside the virus capsid. Therefore, there are some limitations to the VLP-based strategy, including that (1) It is tedious and time-consuming to clone the genes, (2) it is hard to control the encapsulation of the cargo gene into VLP inside host cells or bacteria, and (3) the production yield of the effective VLP is relatively low.

Motivated by viruses and VLPs, researchers have developed recombinant protein or synthetic peptide-based artificial viruses (Figure 2). Among these artificial viruses, our group has pioneered the construction of spherical parapoxvirus-like nanococoons by co-assembling a short synthetic peptide K3C6SPD (Figure 1) with pDNA [81]. K3C6SPD contains 16 amino acids, including three functional segments: (1) a positively charged N-terminal oligolysine, (2) a central β-sheet folding segment, and (3) a C-terminal hydrophilic fragment. K3C6SPD self-assembled into nanofibrils, while K3C6SPD co-assembled with pDNA into an artificial virus (called nanococoon), with the ordered peptide β-sheet nanofibrils wrapping the reporter gene inside. This artificial virus effectively protected DNA from enzymatic digestion and transported DNA into cells. Through structure characterization, we proposed the nanococoon formation mechanism: the electrostatic interaction between the peptide and DNA drove the preorganization of peptide strands along the DNA backbone, followed by peptide assembling into nanofibril and nanofibril-nanofibril association, thereby wrapping DNA into nanococoons. We subsequently investigated how the nanofibril–nanofibril association impacted on nanococoon properties and found that the hydrophobicity of amino acid side chains and the length of the β-sheet-forming segment significantly influenced the morphology and stability of the peptide–DNA co-assemblies [82].

Inspired by filamentous tobacco mosaic virus (TMV), several rod-shaped TMV-like artificial viruses have been constructed (Figure 2). For example, Vries et al. designed a simple chimeric coat protein (>400 amino acids) [75], which co-assembled with pDNA or mRNA into filamentous TMV mimics [75,83]. Inspired by the same TMV model virus, Stupp’s group used the α-helical peptide-spermidine hybrid formulation material to encapsulate linear or circular DNA, leading to the formation of the rod-like artificial virus [84]. Currently, the developed peptide/protein-based artificial viruses are mainly used to condense long nucleic acids (e.g., pDNA or mRNA). For short nucleic acids (such as siRNA), different strategies have been developed. For example, Lee et al. preassembled filamentous nanoribbons with a short peptide, which absorbed siRNA on the surface through electrostatic interaction [85].

These synthetic protein/peptide-based artificial viruses are different from natural viruses and VLPs in two aspects: (1) The “capsid” is formed by the recombinant protein or synthetic peptide, instead of by virus coat proteins, and (2) the encapsulation of nucleic acids is easily controlled and scaled up in test tubes. These artificial viruses also have advantages over polymer/lipid-based nucleic acid carriers, including the following: (1) The origin of the synthetic peptide/protein is more biologically relevant to the native protein; (2) the ordered peptide/protein secondary structures stabilize the whole structure, instead of the non-specific hydrophobic interactions within the disordered or bilayer-structured polyplexes and lipoplexes, respectively; (3) the virus-like assembly process ensures the encapsulation of nucleic acids in the core with good gene protection; and (4) the cooperative formation of artificial viruses allows tunable assembly and disassembly. In this category, several requirements should be met for both DNA and RNA condensation: (1) The peptide/protein has the capability to self-assemble into ordered nanostructures, (2) the peptide/protein should contain a positively charged segment, and (3) a hydrophilic end segment is usually required to maintain the stability of the resulting artificial virus.

## 3. Cellular Internalization of Nucleic Acids

**Figure 3:**
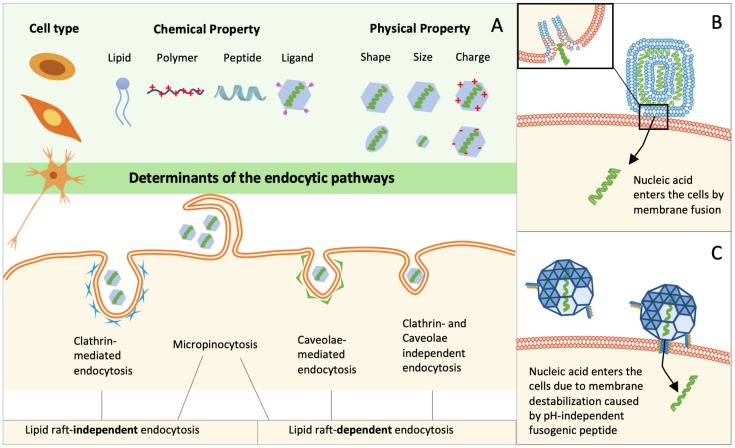
Cellular internalization pathways of nucleic acid carriers. (**A**) Summary of the determinants on the endocytic pathways. Cell types and the chemical and physical properties of formulation materials are all influential parameters of endocytic pathways. Endocytic pathways for nano-sized complexes can be classified into clathrin-mediated endocytosis, caveolae-mediated endocytosis, clathrin- and caveolin- independent endocytosis, and micropinocytosis. This can also be classified into lipid raft-dependent endocytosis and lipid raft-independent endocytosis. (**B**) In a lipoplex delivery system, nucleic acids can directly enter the cytosol by membrane fusion. (**C**) In a peptide/protein-based polyplex delivery system, a pH-independent fusogenic peptide can destabilize the cell membrane, resulting into direct entrance of nucleic acid into cytosol.

Condensed nucleic acids usually enter into cells through endocytosis. The endocytic pathways are categorized into different groups (e.g., clathrin-mediated endocytosis, caveolae-mediated endocytosis, clathrin- and caveolin- independent endocytosis, and micropinocytosis) based on the type of cytoplasmic proteins involved or the composition of the lipid rafts involved (Figure 3) [86]. Therefore, the resulting vesicles are different in lipid composition and/or the surface-exposed proteins. Since the cellular trafficking process of endocytosed complexes is orchestrated by multiple cytoplasmic proteins exposed on the in situ generated vesicles, the different endocytic pathways may influence the fate of nucleic acids, leading to the following possibilities: (1) Different compositions of the surface proteins on the vesicles control the amount of cargo being recycled back to cell membrane, control the binding affinity of the endosome with the motor protein dynein, and also control the acidification time frame of the endosome from neutral to acidic pH; (2) different physical properties (size, shape) of the vesicles influence the trafficking rate toward the nucleus (see Section 6); (3) the endosome maturation process and microtubule-mediated trafficking rate together regulate the spatial/temporal endosomal escape of the genetic cargo. Therefore, the endocytic pathways of nucleic acid complexes are important for genetic cargo to exert their gene transfection functions. The endocytic pathways depends on the physical properties (size, shape, and surface charge) and chemical properties (formulation material chemical properties and surface ligands) of the complexes, as well as the cell types (Figure 3) [87,88]. The influence of the physical properties of complexes has already been reviewed elsewhere [89,90,91]. Here, we mainly discuss the impact of the chemical properties of different formulation materials on the cellular internalization pathways of the corresponding nucleic acid complexes.

### 3.1. Cellular Uptake of Lipoplexes

Lipoplexes are composed of unique lipid molecules, which share similar chemical properties with lipids in cell membranes. Therefore, lipids with special properties may promote cellular internalization through direct membrane fusion (Figure 3B) [92]. The example lipid molecules include the inverted cone-shaped DOPE and the rigid aromatic ring-containing cholesterol, which easily fuse with cell membrane. Recent studies have reported the direct fusion of lipoplexes (DOTAP: DOPE: cholesterol 11:2:7) as the main cellular internalization pathway for siRNA delivery into HeLa cells [93]. Lu et al. also observed the direct fusion of the (lipid: DharmaFECT1) lipoplex for siRNA delivery [94]. However, direct fusion only accounts for the internalization of a small population of lipoplexes, while the majority enters into cells through endocytosis. Lipofectamine-based lipoplexes entered into cells through endocytosis, which resulted in much higher efficiency (~45%) than did adenovirus (~10%) [95]. The molecular shape-match between lipids in lipoplexes and the cell membrane determines the endocytic pathways. Specifically, the lipoplexes containing aromatic-ring-containing cholesterol and/or inverted cone-shaped lipids (DOPE) interact with lipid rafts composed of tightly packed sphingolipids and cholesterol; the lipoplexes containing cylinder-like lipids (DOTAP and DOPC (1,2-*d*ioleoyl-sn-*g*lycero-3-*p*hosphocholine)) interact with fluid-phase domains that have an abundance of cylinder-like unsaturated lipids. Caracciolo et al. observed this correlation by comparing the lipid composition-dependent cellular internalization pathways in Fibroblasts NIH 3T3 cells [96]. Therefore, tailoring the surface of lipoplexes into a patchwork-like plasma membrane is a feasible method to tune the endocytic pathways.

### 3.2. Cellular Uptake of Polymer-Based Polyplexes

The chemical properties of polymers and cell membranes are dramatically different and no direct fusion has been observed for the internalization of polyplexes. They usually enter into cells through endocytosis. They firstly bind with negatively charged glycoproteins such as proteoglycans on the cell membrane; this is followed by the invagination of plasma membrane (Figure 3A). So far, no direct correlation between the properties of polymers on cellular internalization pathways has been established. The most popular strategy to direct endocytic pathways of polyplexes is through surface-modified ligands. For example, folic acid (FL) guides the FL-PEI/pDNA polyplexes to enter into cells through clathrin-independent endocytosis, while transferrin (TF) promotes the cellular internalization of TF-PEI/pDNA polyplexes through clathrin-mediated endocytosis [97]. The length of surface-exposed oligo-arginines also influences the endocytic pathways of polyplexes. Through investigation of 1-, 4- and 8-residue oligo-arginines decorated PEG-PCL (polycaprolactone) nanoparticles (denoted as R1PECL, R4PECL, and R8PECL) [98], we have discovered that non-modified and R1PECL particles entered into cells via clathrin-mediated endocytosis, R4PECL particles via lipid-raft dependent endocytosis, and R8PECL particles via both lipid raft-dependent endocytosis and macropinocytosis. We also demonstrated that the surface ligand density did not influence the endocytic pathways, but did influence the number of internalized polyplexes [98]. In addition, ligand modification has also been applied to enhance the cellular internalization of polyplexes through receptor-mediated endocytosis. For example, the epidermal growth factor receptor-modified polyplexes increased the cellular uptake of the cargo from 20% to 50% [99]; and decoration of CPPs on the polyplexes showed 10-fold cellular uptake improvement [100]. Therefore, for polyplexes, the choice of the surface ligands is very important for the internalization of delivered nucleic acids.

### 3.3. Cellular Uptake of Peptide-Based Polyplexes and Artificial Viruses

Some peptides, due to their biological potential for adopting α-helical structure, can destabilize cell membranes through membrane insertion. This group of peptides (such as MPG, CADY, and penetratin) has the chance to directly translocate nucleic acid cargoes into cells through membrane destabilization (Figure 3C) [101]. By systematic analysis of the physical and structural properties of these peptides, Divita et al. proposed a cellular uptake model for these polyplexes [102]. MPG/nucleic acid polyplexes bound with proteoglycans on the cell membrane through electrostatic interaction, which activated the intracellular signal. Then, the polyplexes interacted with phospholipids, followed by the peptide insertion into the cell membrane. This process was accompanied by a peptide conformational transition and membrane potential change, which led to the formation of a voltage-dependent pore-like structure, allowing the direct translocation of the polyplexes into the cytoplasm [67,103]. As for peptide-based polyplexes without α-helical peptides, they usually enter into cells through endocytosis. In our laboratory, we have developed a peptide-based virus mimic that contains a β-sheet nanofibril “capsid”. This artificial virus entered into cells via endocytosis [82]. By changing the peptide sequence, spherical peptide/nucleic acid polyplexes switched their morphology to long filamentous-like complexes, which mainly attached on the cell surface with low uptake efficiency (unpublished data). Therefore, the secondary structure and the self-assembled morphology of the peptides are important parameters to determine internalization pathways.

## 4. Endosomal Escape of Nucleic Acids

### 4.1. Environmental Changes during the Endosome Maturation Process

**Figure 4:**
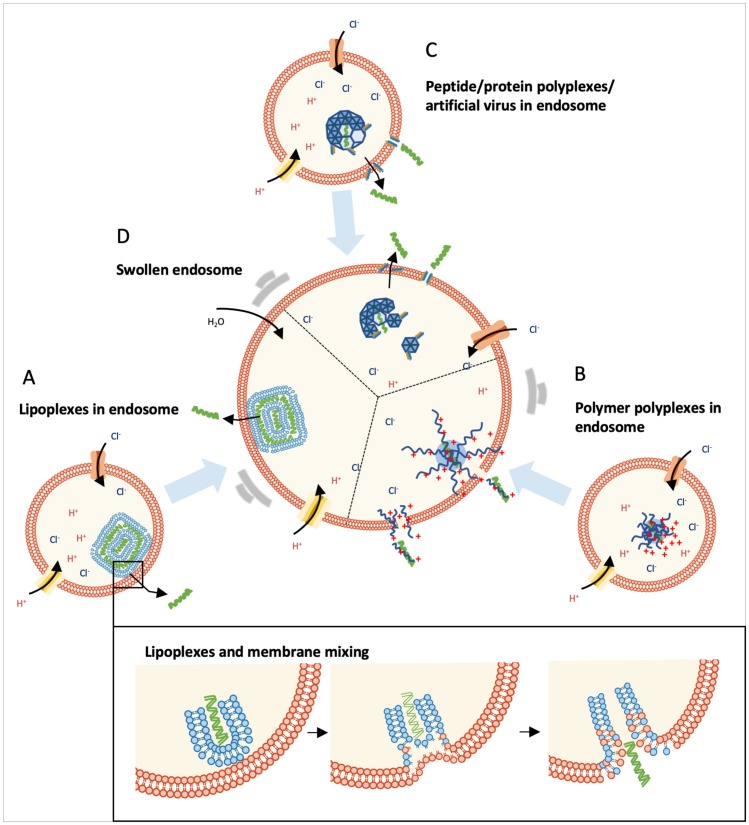
Summary of the formulation material property-dependent endosomal escaping mechanisms. (**A**) In a lipoplex delivery system, nucleic acids escape from the endosome via lipid mixing. One widely accepted model, the pore formation via flip-flop of the endosome membrane, is described in the bottom zoom-in picture. (**B**) In a polyplex delivery system, the endosomal escape of nucleic acids is through membrane destabilization by polyplexes or free polymers. (**C**) In a peptide/protein-based delivery system, the endosomal escape of nucleic acids happens via different mechanisms, including membrane destabilization and pore formation. (**D**) Endosomes containing lipoplexes, polyplexes or peptide/protein polyplexes with buffering capability swell through the protonation of secondary or tertiary amines, followed by the influx of the counter ions and water. The swollen endosome promotes efficient endosomal escape of genetic cargoes through lipid-mediated slow release or polymer-mediated fast squirting of nucleic acids into cytosol, respectively.

Vesicles formed in situ during endocytosis are invaginated in a plasma membrane that encloses nucleic acid complexes. These vesicles produced from different pathways follow a similar endosome-lysosome maturation process, though the time points to join this process may be different [104,105]. This maturation process is induced by several Rab GTPases [106] and the phosphatidylinositols (PIs) in the endosome vesicle bilayer [107]. This endosome maturation process is accompanied by a pH drop from neutral to slightly acidic (in early endosomes, pH6.5 to 6.0; in late endosomes, pH5.5 to 5.0; and in lysosomes, around pH5.0 to 4.5) [104]. Since endosomes and cell membranes have the same lipid components, the complex–endosome interaction during the endosomal escape process shares a similar mechanism with that of the complex–cell membrane interaction during the internalization process. The difference is the environmental pH for both processes (acidic for endosomal escape vs. neutral for cellular internalization). Most synthetic nucleic acid complexes enter into cells through endocytosis and end up in lysosomes for enzymatic digestion. Therefore, the endosomal escape of nucleic acids is critical to enhance their active population and the overall gene transfection/inhibition efficiency. Different endosomal escaping mechanisms, including pH-triggered membrane disruption, pore formation, and fusion with the endosomal membrane, are discussed and summarized (Figure 4) based on the chemical properties of different formulation materials.

### 4.2. Lipid-Mediated Endosomal Escape

Since the endosome and cell membrane share the same lipid components, a similar membrane fusion hypothesis is applied to the lipid-mediated endosomal escape of lipoplexes (Figure 4A) [108]. As we discussed in Section 3, the special molecular properties of DOPE and cholesterol also allow the endosomal escape of the cargoes through direct fusion with endosome membranes. Therefore, fusogenic lipid DOPE has been extensively exploited in lipoplex formulation [109], and also in polyplex formulations (PEI/siRNA) to facilitate endosomal escape [110]. Similarly, lipoplex formulations containing cholesterol (DC-cholesterol/DOPE/DNA) showed successful endosomal escape, whereas the control one (DOTAP/DOPC/DNA) without cholesterol was trapped [111]. In addition, the structure of lipid tails influences their endosomal escaping capability. Various approaches (including shortening the lipid tail, altering the symmetry of the tail, switching the linear tail to a branched tail, or decreasing the saturation degree of the tail) have been developed to promote endosomal escape by either decreasing the transient temperature or enhancing the fluidity of the bilayers and lipid mixing capacity [41].

The pH-sensitive lipids that contain amino groups with pKa 5 to 6 facilitated endosomal escape through a sponge effect (Figure 4D) [31]. The pH-sensitive lipids are protonated upon an environmental change from a neutral to acidic pH, which is accompanied by an influx of counter ions and water. This process leads to an increase of osmotic pressure, which promotes endosomal escape of the lipoplexes through membrane destabilization. Jayaraman et al. discovered that the lipoplexes containing a pH-sensitive lipid enhanced the endosomal escape of the cargoes [112]. Lipofectamine, a “gold standard” representative of lipoplexes, contains the pH-sensitive lipid DOSPA. DOSPA carries primary, secondary, and tertiary amines, and the secondary amine shows the proton sponge effect, which enhances the endosomal escape efficiency of lipoplexes [47].

### 4.3. Polymer-Mediated Endosomal Escape

The polyplexes containing secondary amino groups (such as the ones in PEI and poly(amidoamine)) are generally believed to escape from the endosome through a combination of the sponge effect and umbrella effect (Figure 4B,D) [51]. PEI has two forms, including linear PEI (lPEI) and branched PEI (bPEI) (Figure 1). Linear PEI, which contains secondary amino groups, has contributed significantly to another “gold standard” in the field of nucleic acid delivery. It absorbs protons efficiently in the endosome during the endosome maturation process [113], which is accompanied by an increase in osmotic pressure inside the endosome due to the influx of the counter ions and water [47,114]. At the same time, charge repulsion between the protonated amines causes the expansion of PEI polyplexes; this is known as the umbrella effect [114,115]. The loose PEI polyplexes interact and destabilize the endosome membrane, promoting endosomal escape of polyplexes. Recent live cell imaging studies demonstrated that local membrane destabilization or local pore formation was the sponge effect-mediated endosomal escape of polyplexes. Live cell confocal microscope imaging showed that the polyplexes ejected nucleic acids into cytosol from particular regions of the endosome [116]. Therefore, the endosomal escaping capability of polyplexes depends on the overall effect of the increase in osmotic pressure induced by protonated polymers and the membrane destabilization caused by polymer-membrane interaction. Polyplexes enter into cells via diverse pathways, followed by different acidification rates. This might explain why polyplexes based on the same formulation material display different endosomal escaping capabilities and gene transfection efficiencies in different cell lines.

### 4.4. Peptide-Mediated Endosomal Escape

Short peptides promote the endosomal escape of nucleic acids through different mechanisms, including the sponge effect, the fusogenic effect, and pH-sensitive membrane disruption (Figure 4C,D). These peptides enhance the endosomal escape of the corresponding complexes when they function either as nucleic acid complexation formulation material or as the surface ligands of lipoplexes and polyplexes. Histidine (pKa of 6.0)-containing peptides show a proton-buffering capacity, which enhances endosomal escape through the sponge effect (Figure 4D) [117,118]. By conjugating histidine with chitosan through cleavable disulfide bonds, Kai et al. developed histidine-modified chitosan/pDNA polyplexes with enhanced endosomal escape [119]. Fusogenic peptides containing pH-sensitive amino acids (such as Glu (E) and His (H)) show a conformation switch from random-coil to the **α**-helix upon a pH drop in the endosome [120]. The **α**-helical peptides interact and destabilize the endosome membrane, achieving endosomal escape of nucleic acids [121]. One of the representative fusogenic peptides is HA2 (GLFGAIAGFIENGWEGMIDGWYG) (Figure 1), which is derived from the hemagglutinin of the influenza virus. Inspired by HA2, several mutations and fusogenic peptides have been developed, including INF7 (GLFEAIEGFIENGWEGMIWDYG), E5 (GLFEAIAEFIEGGWEGLIEG) and GALA (WEAALAEALAEALAEHLAEALAEALEALAA) [122,123,124]. GALA has been extensively applied to different nucleic acid delivery systems to facilitate endosomal escape, such as polymer systems [125] and lipid systems [126]. Some membrane-disruptive peptides, being deactivated through side-chain protection with pH-sensitive groups, can be reactivated under conditions of low pH. For example, carboxydimethylmaleic (CDM)-modified melittin showed minimal cell membrane disruption capability, while the cleavage of CDM in an acidic environment in the endosome restored the membrane-penetrating activity of melittin, enhancing its endosomal escape efficiency (Figure 4C) [127].

## 5. Nucleic Acid Release from the Carriers

**Figure 5:**
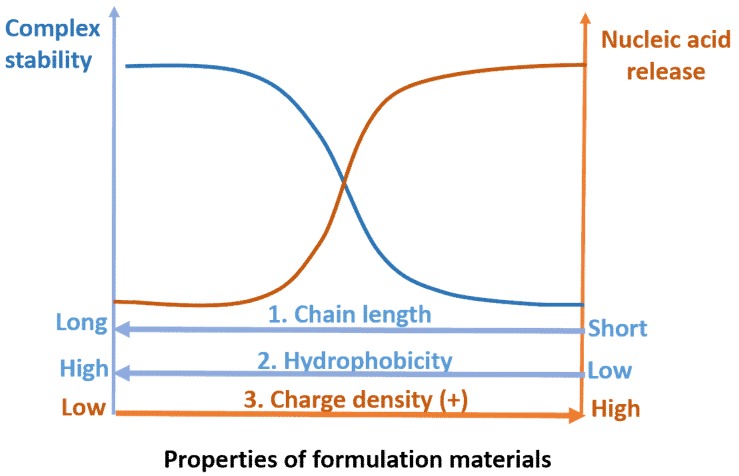
Balance of the stability of polyplexes and nucleic acid release by varying formulation material properties—specifically, elongation of the polymer chain length, enhancement of the hydrophobicity, and decrease of the charge density of the molecules will enhance the complex stability but lower the chances of nucleic acid release. Otherwise, the nucleic acids are easily released, but the stability of the complexes is compromised.

After endosomal escape, the nucleic acids (such as mRNA and siRNA) that function in cytoplasm should be released from the carriers in order to augment or inhibit protein translation by binding with cytoplasmic machinery. However, the nucleic acids (such as pDNA) that function in the nucleus should be further protected before they reach or enter the nucleus [128]. Nucleic acid release is reversible by the condensation process (Figure 5), so the capability of nucleic acid released from different carriers is determined by the chemical and physical properties of different formulation materials.

As we discussed in Section 4, lipids in lipoplexes interact with lipids in the endosome membrane. This causes membrane fusion or membrane disruption. This process allows endosomal escape as well as nucleic acid release from lipoplexes. Immediate spreading of siRNA from the endosome into the whole cytoplasm was observed in live cell imaging of lipoplex delivery systems [129,130]. Therefore, lipoplexes are particularly suitable to deliver nucleic acids, which function in cytoplasm.

Polyplexes are much more stable than lipoplexes, due to their long polymer chains. Moreover, polyplexes usually escape from the endosome through the interaction between the endosome membrane and part of the polymer chain, which allows the intact polyplexes to get into the cytoplasm. Early studies have indicated that nucleic acids are usually released from these polyplexes through anionic exchange with the genomic DNA [131] and RNA [132]. However, the efficiency of nucleic acid release from the carriers is uncontrollable if the release merely counts on the polyanionic exchange with endogenous biopolymers. Therefore, different strategies have been studied to regulate cellular nucleic acid release, which can be achieved by fine-tuning the charge density and chain length (Figure 5) [128].

Polymer charge density influences the binding intensity of polymer and nucleic acids. For example, partial covering of the positive charges (the secondary (23%) and primary (43%) amines) of branched PEI (25kDa) by acylation improved the dissociation ability of nucleic acids from the polyplexes [133,134]. Introduction of the negatively charged carboxylic acid groups on the side chain of linear PEI led to the enhancement of DNA release from polyplexes [135]. Polymer length or molecular weight also controls nucleic acid release. Polymers of low molecular weight show easier nucleic acid release [136], while longer polymers hamper nucleic acid release through stronger interactions with nucleic acids and/or the formation of a knot-like structure with nucleic acids [137]. Therefore, the intracellular glutathione (GSH)-triggered polymer chain length decrease has been frequently used to achieve nucleic acid release [138]. Degradable redox-responsive polymer cysteine-based poly(disulfide amide) (PDSA) realized a rapid GSH-triggered gene release [139]. Therefore, the responsive nucleic acids release from polyplexes adds value to nucleic acids delivery with polymeric formulation materials.

## 6. Nuclear Trafficking of DNA

### 6.1. Challenges of Free DNA in Cytoplasm

Viscous cytoplasm presents a major challenge for DNA to move toward the nucleus. The diffusion of DNA larger than 2 kbp in cytoplasm is highly restricted [140]. Previous studies have demonstrated that it is much harder for naked plasmids in cytoplasm to be expressed than those injected into the nucleus [141]. Therefore, intracellular trafficking towards the nucleus is critical for DNA to accumulate in the perinuclear area. Enzymatic digestion is another challenge for free DNA in cytoplasm. Previous studies have shown that a microinjection of naked DNA into cytoplasm led to no DNA transfection, while the injected PEI-polyplexes containing the same amount of DNA showed a high degree of transfection [12]. Free DNA released from the carriers is easily digested by cytoplasmic enzymes [142], so further protection during the nuclear trafficking process is required from the synthetic carriers. Through PCR (polymerase chain reaction) and electron microscopy analysis, it was discovered that of about 2000 to 100,000 plasmid copies delivered into cytoplasm by polyplexes and lipoplexes, only 1% to 10% of these plasmid copies were delivered into the nucleus [143]. Therefore, nuclear trafficking and nuclear entry are believed to be rate-limiting steps for efficient gene delivery and transfection. In the past 20 years, enormous efforts have been put into elucidating mechanisms for the nuclear trafficking and nuclear import of plasmid, which are important for us to develop efficient strategies for DNA delivery.

### 6.2. Vesicle and Ligand-Guided Active Nuclear Trafficking of DNA

**Figure 6:**
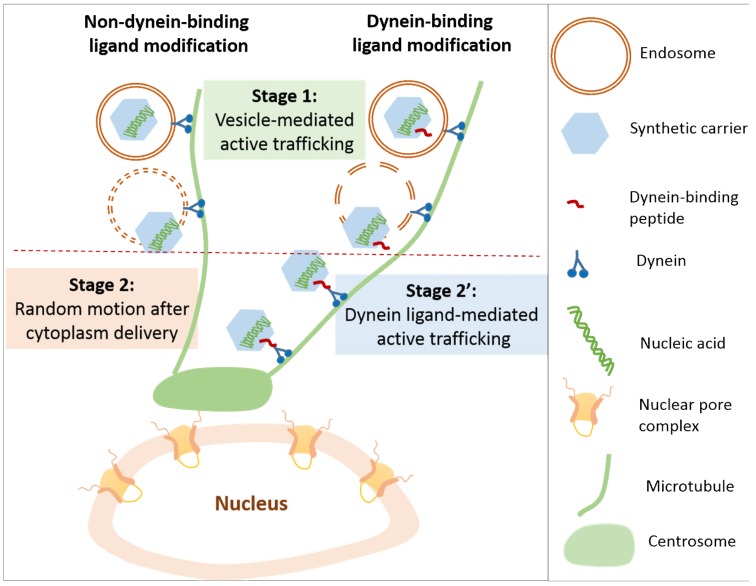
Nuclear trafficking pathways. For the complexes without dynein-binding ligand modification, the directed trafficking toward the nucleus occurs through vesicle mediation. Once the complexes are released from the endosome, they will either be stuck or diffuse within a limited space. For the complexes modified with dynein-binding peptides, the directed trafficking is mediated by vesicles and dynein-binding peptides. Accumulation around the nucleus will be achieved.

Lipoplexes without ligand modification showed directed trafficking toward the nucleus along microtubules. With three-dimensional (3D) Single-Particle Tracking (SPT) techniques, Caracciolo et al. observed the directed motion of lipoplexes (DOTAP/DOPC/DC-Cholesterol/DOPE/pDNA)) inside cytoplasm [13]. This is consistent with the observations by Ondrej et al. and Sauer et al. that lipoplexes bound to, and moved along, microtubules [144,145]. By investigating the mechanical dynamics of DNA lipoplexes in live cells through bio-imaging approaches [146], Jones et al. discovered that the motion rate of lipoplexes within cytoplasm was cellular location-dependent, but cargo (DNA) size-independent (21 bp to 5.5 kbp). In these studies, endocytosed vesicles (such as endosome) enclosing lipoplexes accounted for the directed nuclear trafficking (Figure 6) [147].

In later studies, Caracciolo et al. compared lipofectamine-based lipoplexes with high transfection capabilities (high-lipoplexes) to a control (DOTAP/DOPC/DNA) with low transfection efficiency (control) through a combination of live cell imaging, single-particle tracking microscopy, and quantitative transfection-efficiency assays [148]. They observed that the control moved mainly through active trafficking along microtubules, whereas the high-lipoplexes mainly trafficked towards the nucleus (64.3%) through random Brownian diffusion. The different motion of the high-lipoplexes and the control may be attributed to their different endosomal escaping capabilities and time frames of escape. The control being trapped inside the endosome/lysosome may explain why it shows low transfection efficiency.

For the intracellular trafficking of PEI polyplexes, influenza virus-like three-phase processes were observed, including 1) slow drift movement, followed by 2) confined diffusion with increased velocity, and finally 3) fast active movement along the microtubule [99]. Using the Multiple Particle Tracking (MPT) technique, Suh et al. observed the microtubule-directed active transportation of PEI polyplexes to the perinuclear area in COS-7 cells [149]. Through Raster Image Correlation Spectroscopy (RICS) and image-Means Square Displacement (iMSD) analysis, Jones et al. observed a similar population of PEI polyplexes with active and non-active motion.

The directed motion and random diffusion of both lipoplexes and polyplexes in cytoplasm was observed with the single particle trafficking technique. A reasonable explanation is that the directed motion observed for the bare lipoplexes and polyplexes may be attributed to vesicular transport and the random diffusion is for the particles that escaped from the endosome (Figure 6) [150,151]. Confocal images showed that more than 90% of lipoplexes and polyplexes had directed motion when they co-localized with the lysosome. Once they escaped from the lysosome, the directed motion switched to random diffusion in cytoplasm. Therefore, the timing of endosomal escape is critical for the perinuclear accumulation of polyplexes and lipoplexes, leading to different degrees of transgene expression.

Since active trafficking along microtubules is mainly mediated by intracellular vesicles, the different vesicles generated through different internalization pathways significantly influence the transportation rate. Specifically, the vesicles generated through the clathrin-mediated pathway showed directed motion with an average velocity of 0.7 μm/s [152]. A similar velocity value (0.65 μm/s) was observed for PEI polyplexes in HuH-7 cells [150]. The velocity of ~0.13 μm/s was observed for polymeric nanoparticles internalized through a clathrin- and caveolae-independent pathway. Micropinocytozed R8-modified lipoplexes showed a slower transportation rate of 0.21 ± 0.19 μm/s [151]. In addition, PEG-PLL polyplexes, internalized through a caveolae-dependent pathway, had a directed trafficking rate between 0.09 and 0.11 μm/s [153]. This vesicle type-dependent movement may be attributed to their surface-exposed proteins, which have a different binding affinity with the motor protein, dynein. However, the different cell lines used in these studies may affect the formation of vesicles and the adaptor proteins displayed on these vesicles, which significantly influences vesicle-mediated trafficking. In these studies, the final destination of different polyplexes/lipoplexes was not defined, so it will be meaningful to systematically study and correlate the type of polyplexes/lipoplexes with their cellular internalization pathway, intracellular trafficking rate, and final destination.

Microtubule-mediated active trafficking of cellular vesicles (such as endosomes), as well as viruses (such as adenovirus and herpes simplex virus) inspires the investigation of peptide ligand-mediated active nuclear trafficking [154,155,156]. For example, decoration of dynein light chain (LC8)-associated peptides on lipoplexes/polyplexes mimics the active trafficking of viruses (Figure 6). Specifically, CPP octa-arginine (R8) and African swine fever virus protein p54-derived dynein interaction peptide-modified lipoplexes achieved directed trafficking independent of endosome vesicles, while the control lipoplexes without a dynein-binding protein only showed directed trafficking in the presence of endosome vesicles [157].

## 7. Nuclear Entry of DNA

### 7.1. Passive and Active Nuclear Accumulation of DNA

**Figure 7:**
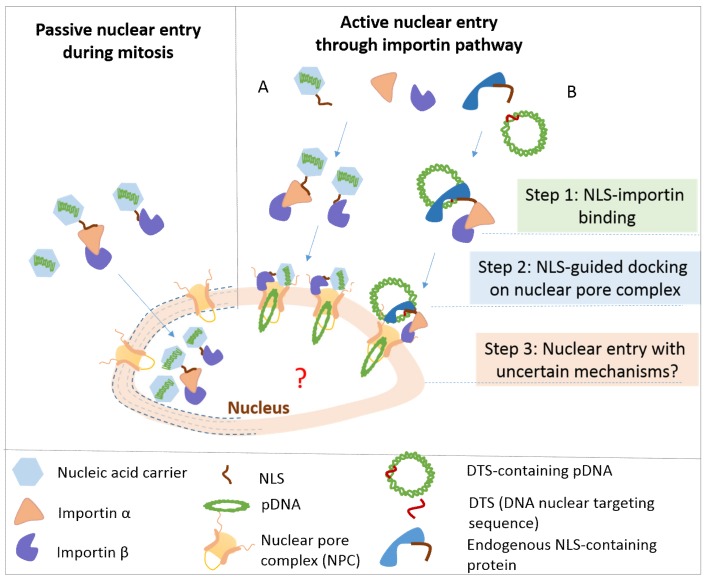
Two different nuclear entry strategies: passive vs active pathways. For dividing cells, all the nucleic acid complexes near the nucleus have a chance to enter into the nucleus during mitosis. However, for non-dividing cells, active docking on the nuclear pore complex (NPC) through an importin pathway is required. In case A, complexes modified with a nuclear localized signal (NLS) peptide can bind with importin β through importin α or directly, and importin β will drive the docking on NPC. In case B, the plasmid with DTS (DNA-nuclear targeting sequence) can specifically bind with an endogenous NLS-containing protein (such as transcription factors) that will bind with importin β and lead to active docking on NPC. As for the following step of nuclear entry of DNA or nuclear entry of an intact complex, its mechanism remains to be addressed. Reproduced with permission from spring nature [165].

For DNA delivery, the nuclear membrane is one of the subcellular barriers that significantly impacts the overall gene transfection efficiency. The nuclear pore complex (NPC) is a large protein complex that forms nuclear pores on the nuclear envelope, allowing the import and export of macromolecules. Molecules of a size smaller than 9 nm freely diffuse into/out of the nucleus, while molecules with a size range of 9 nm to 39 nm require a signal-mediated process for nuclear entry [17]. The nuclear entry of long pDNA through NPC is restricted and passive entry is believed to be the primary pathway [158]. In another words, the breakdown of the nuclear envelope during mitosis allows the entry of any DNA that is near the nucleus (Figure 7). This may explain why many growth-arrested cells (such as primary cells and neurons) are difficult to be transfected [159].

Lipoplexes and polyplexes did show a certain degree of transfection in non-dividing cells [160]. For these cases, it is generally believed that lipoplexes and polyplexes promote nuclear entry through fusion with the nuclear envelope [161] or permeation into the nuclear membrane [162], respectively. Szoka et al. investigated the nuclear entry of lipoplexes and polyplexes with the commercial model reagents lipofectamine and PEI. Through standardized quantitative PCR assay, they discovered that lipoplexes and polyplexes delivered similar amount of plasmids from extracellular media into the nucleus [143]. With confocal laser microscopy, Harashima et al. observed the fast nuclear localization of DNA by lipoplexes within 0.5 to 1 hr [161]. They quantified the intracellular distribution of DNA and observed that of the total cellular internalized DNA, 13.5% was accumulated inside the nucleus within one hour. The intact PEI/DNA polyplexes were observed in the nucleus [163]. Through intracellular trafficking of fluorophore-labeled PEI/DNA polyplexes, Mikos’s group discovered that most of the polyplexes were trapped inside the endosome right after endocytosis and accumulated in the nucleus after four hours [164]. They speculated that the positively charged PEI interacted with negatively charged lipids, and these bound lipids facilitated the nuclear entry of PEI/DNA polyplexes. The exact mechanism remains to be explored.

### 7.2. NLS-Mediated Active Nuclear Entry of DNA

Nuclear localization signals (NLSs) are a group of basic residue-rich short peptides which mediate active nuclear entry of protein and DNA. One of the well-studied NLSs is PKKKRKV (Figure 1), derived from virus SV40 large T-antigen. NLS-mediated nuclear entry is through the importin pathway (Figure 7) and includes the following steps: (1) NLS directly binds with importin β or through the adaptor protein importin α; (2) importin β guides the docking of the NLS-importin complex on NPC, leading to nuclear entry through an unknown mechanism [165,166]. In mammalian cells, there are 6 importin α and 20 importin β protein isoforms. The combination of these importins facilitates the translocation of different cytoplasmic cargoes into the nucleus.

Based on the mechanism of the importin pathway for nuclear entry, the introduction of NLS to the complex allows DNA to be recognized by importin. Therefore, the short NLSs derived from different proteins have been explored for nuclear entry through (1) non-covalent electrostatic interaction with DNA and (2) covalent conjugation with delivery formulation materials, such as polymers and lipids. For example, SV40 NLS-conjugated lipoplexes showed a threefold increase of nuclear accumulation [167] and enhanced the transgene expression ten- to eleven-fold [168]. The conjugation of NLS to PEI/cyclodextrin/DNA polyplexes led to enhancement of transgene expression in both dividing and non-dividing cells [169]. Through confocal imaging, Chu et al. observed that NLS-polyplexes entered into the nucleus in six hours, while the control polyplexes only accumulated in the perinuclear region in the cytoplasm [169]. This data demonstrates the role of NLS for nuclear delivery and is consistent with the enhancement of gene transfection [158].

The binding of NLS with importin requires the exposure of NLS on the surface, so the nuclear delivery efficiency of NLS/DNA complexes depends on the number of NLS exposed on the complex surface. If NLS is used as the DNA condensation agent, the complexation with DNA may bury an NLS with a low importin-binding capability. In previous studies, the addition of NLS did show improved gene transfection, but it is not clear whether the improvement of gene transfection resulted from the enhancement of nuclear entry, since nuclear entry has seldom been directly characterized. Therefore, in future studies, it will be necessary to define the increase of the amount of DNA entering into the nucleus after the addition of NLS. Such quantified studies can assess the capability of NLS to facilitate the nuclear entry of DNA.

Besides the short NLSs, endogenous proteins (such as transcription factors and histone proteins) contain an NLS functional segment. These proteins specifically recognize nucleic acids containing a special DNA nuclear targeting sequence (DTS) and facilitate active nuclear entry through an importin pathway (Figure 7) [170]. For example, a short DTS of 72 bp, derived from SV40 plasmid and bound with NLS-containing transcription factors, mediated quick nuclear entry of the plasmid. Besides DTS, the plasmid-containing necrosis factor-α NF-κB-binding nucleic acid sequence could actively enter into the nucleus [163]. The proteomics study identified several NLS-containing proteins, including histone H2B, a ubiquitous nucleoside diphosphate kinase (NM23-H2), and the homeobox transcription factor, Chx10 [171]. Therefore, conjugation of NLS on the delivery vehicles and/or addition of an NLS-binding nucleic acid segment into the plasmid are two strategies to promote active docking on the nuclear pore complex.

### 7.3. Other Strategy-Facilitated Nuclear Entry of DNA

Besides the importin pathway, the cell surface nucleolin, a ubiquitous eukaryotic protein, shuttled polyplexes from the cell membrane to the nucleus in an endocytosis-independent manner [172]. Davis et al. discovered that the PEG-PLL/DNA polyplexes directly bound with the cell surface receptor nucleolin and traveled together towards the nucleus. Their later studies demonstrated that these polyplexes entered into cells through lipid raft-mediated entry [173]. In addition, manipulation of the nuclear complex pore size through receptor interaction is able to facilitate nuclear entry of polyplexes. Fan et al. discovered that cytoplasmic glucocorticoid receptor-specific ligand-modified hyaluronic acid/PEI/dexamethasome/DNA polyplexes enhanced nuclear entry [174] through a ligand-receptor-mediated nuclear pore size increase (up to 300 nm) [175].

## 8. Summary and Prospects

Natural and synthetic formulation materials play significant roles in facilitating exogenous nucleic acids to function inside cells. Lipids, polymers, and peptides are the most extensively studied formulation materials. The charge attraction and hydrophobic interaction between these formulation materials and nucleic acids efficiently condenses the macromolecular nucleic acids into nano-sized particles, with nucleic acids being protected within the formulation material matrix. Three kinds of formulation materials have distinct condensation propensities for nucleic acids, resulting in lipoplexes, polyplexes, and peptide-based polyplexes/artificial viruses. These various properties induce different intracellular trafficking routes and cellular fates of nucleic acids. Understanding how the properties of different formulation materials contribute to this process can guide us to develop safe and efficient carriers for gene therapy in the future. Though a direct correlation has not been established from individual studies, we summarize the extensive studies of well-studied formulation materials in this review and draw some links between the properties of the formulation materials and their function for intracellular nucleic acid delivery (Table 1) in order to inspire next-generation gene delivery systems.

Lipids are small molecules that share similar chemical structures as molecules in the cellular/subcellular membrane. This unique chemical property allows lipids to facilitate delivery of nucleic acids through interacting/fusing with plasma, with the endosome and/or with the nuclear membrane. However, their small molecular structure compromises the stability of the resulting lipoplexes. The low stability of these lipoplexes and their interaction with plasma or endosome membrane allow nucleic acids to easily be released from lipoplexes during cellular entry or endosomal escape processes. This is advantageous for the delivery of nucleic acids that function in cytoplasm. However, the ease of nucleic acid release compromises the gene protection for the delivery of nucleic acids, which function in the nucleus. Polymers, due to their high molecular weight, form stable polyplexes. However, the macromolecular structure of polymers makes it difficult for them to interact with lipid membranes (including the plasma, endosome, and nuclear membranes), so protonation of amino groups is critical to induce endosomal escape through the sponge effect and membrane destabilization. Moreover, the high stability of polyplexes poses a challenge for intracellular cargo release from the carriers. Therefore, a responsive molecular property switch (such as pH-triggered or redox-triggered polymer chain length shortening or the polymer chain charge balance change) allows the smart control of nucleic acid release from polyplex delivery systems. For the nuclear delivery of DNA, nuclear trafficking and nuclear entry are big challenges for both lipoplexes and polyplexes. However, peptides are biopolymers, and their biological origin renders them different from synthetic polymers and lipids, including (1) the formation of artificial viruses with an ordered “capsid”-like structure, (2) facilitating endosomal escape, and (3) directing nuclear trafficking and nuclear entry.

**Figure 8:**
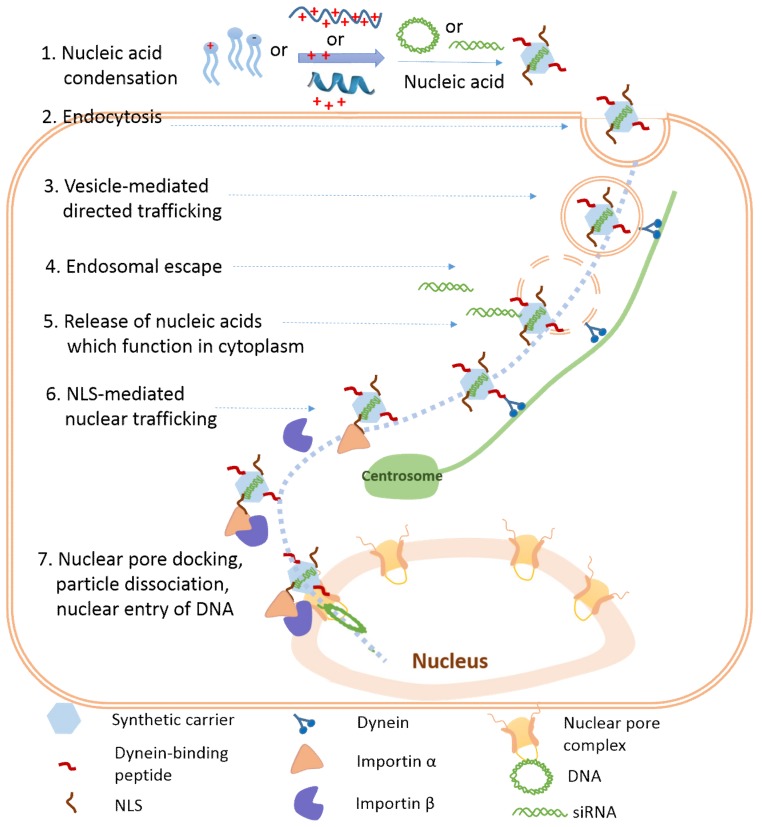
Summary of the formulation material-based nucleic acid delivery to the nucleus. Seven steps are listed from nucleic acid condensation to nuclear entry. The ideal carriers are proposed, which include dynein-binding peptides for directed nuclear trafficking and NLS-modification for nuclear pore complex docking through an importin pathway, as well as the efficient “capsid” uncoating and active nuclear entry of DNA (with uncertain mechanisms).

Given the diversity of viruses (including both naked viruses and enveloped ones), a combination of different formulation materials is one promising approach to develop multifunctional artificial viruses (Figure 8). Many short functional peptides have already been identified from viruses and endogenous proteins, which gives us the opportunity to improve the functions of synthetic artificial viruses. To develop safe and efficient gene delivery carriers is vital for gene therapy. Currently, there is still a big gap between translational studies and the clinical applications of different nucleic acid delivery vectors. Understanding the correlation between the properties of different formulation materials and their nucleic acid delivery capabilities paves the way for next-generation gene delivery vehicles.

## Figures and Tables

**Table 1:** Summary of the representative studies discussed in this review.

Formulation Material	Experimental Nucleic Acids	Experimental Cell Type	Size (nm)	Surface Charge (mV)	Ref.
**Lipid**					
DOTAP/DOPE/cholesterol	siRNA	BSC-40; 293FT; HeLa cells	100–200	N/A	[92]
DOTAP/DOPE/cholesterol; PEI	siRNA	HeLa cells	468 ± 19; 209 ± 17	N/A	[93]
DC-Cholesterol/DOPE	pDNA: luciferase	CHO cells	60–70	58.4–64.7	[94]
DC-cholesterol/DOPE;DOPC/DOTAP; DC-cholesterol/DOPE/DOTAP/DOPC	pDNA: luciferase	NIH 3T3 cells	180 ± 2; 234 ± 4; 205 ± 2	48.3 ± 1.2; 43.3 ± 1.5; 46.7 ± 1.2	[96]
Lipofectamine Plus	pDNA: luciferase	A549 cells	N/A	N/A	[95]
**Polymer**					
PEI (disulfide cross-linked)	pDNA: luciferase	HEK293 T; HeLa cells	~200	~20	[52]
POCG-PEG-PEI polymer	DNA; siRNA; miRNA	MCF-7; C2C12	151; 172; 245	~30	[54]
MPC/Ad-SS-PEG	pDNA: GFP	Hep G2 cells; HeLa cells; SKOV-3 cells; PC-3 cells	100–200	0.5–5	[55]
PEG-PEI (1.8 kDa) (PEI amines are modified with aromatic rings)	pDNA: luciferase mRNA: luciferase siRNA: anti-luciferase	HeLa cells; U87 cells	180–240	N/A	[58]
PEI-stearic acid copolymer	mRNA: ACRA mRNA: HIV-1 gag antigen	DC 2.4 cells	117.77 ± 3.894	N/A	[59]
Folic acid-PEI; transferrin-PEI	pDNA: luciferase	HeLa cells	N/A	N/A	[97]
mPEG-PCL; R-PEG-PCL; RRRR-PEG-PCL; RRRRRRRR-PEG-PCL	N/A	HeLa cells	80–110	20–40	[98]
EGF-PEG-PEI	DNA	HuH7 cells	266 ± 26	N/A	[99]
**Peptide/protein**					
RALA (WEARLARALARALARHLARALARALRACEA)	mRNA: GFP mRNA: ovalbumin	DC2.4 cells	89(N/P 5); 91(N/P 10)	14.6(N/P 5); 26.3(N/P 10)	[65]
MPG-8-cholesterol (MPG-8: *β*-AFLGWLGAWGTMGWSPKKKRK-Cya)	siRNA: Cyc-B1	HS68; HeLa; PC-3; MCF-7; SCK3-Her2 cells	120 ± 50	16 ± 3	[68]
(CRR)2KRRC and (CHH)2KHHC cross-linked peptide	pDNA: p53	NIH3T3 cells; HeLa cells	~164; ~172	~30; ~20	[69]
Virus-like particle (VLP) (Vesicular stomatitis virus and Archeoglobus fulgidus-based)	mRNA: GFP	HEK293T cells; THP-1 cells; Human iPS cells	N/A	N/A	[76]
VLP (neurotropic JC polyomavirus: JCPyV))	pDNA: suicide gene (HSV-TK)	U87-MG cells	N/A	N/A	[77]
VLP (JCPyV))	pDNA: suicide gene (HSV-TK)	Toledo and HT cells; SU-DHL-2 cells	N/A	N/A	[78]
VLP (JCPyV))	pDNA: suicide gene (pSPB-tk)	A549 cell; H460 cell	N/A	N/A	[79]
VLP (JCPyV))	pDNA: suicide gene, (HSV-TK)	COLO-320 HSR cell	N/A	N/A	[80]
Self-assembled peptide: K3C6SPD (KKKC6-WLVFFAQQ-GSPD)	pDNA: GFP	Hek293 cells	~70	25	[81,82]
K12-(GAGAGAGQ)10-407-amino-acid hydrophilic random coil	mRNA: GFP and luciferase; pDNA: YFP	Hek293 cells; HeLa cells	~150 (average length)	-5	[75,83]
Spermine-Coiled-coil peptide-PEG (Coiled coil peptide: REGVAKALRAVANALHYNASALEEVADALQKVKM)	N/A	N/A	N/A	N/A	[84]
Glucose-GSGSGSKKKKKKKKGGSGGSWKWEWKWEWKWEWG	siRNA: GFP	HeLa cells	~70	~0	[85]
CPP-based polyplexes shelled with polysaccharide (CPP: RRRRRRRR)	pDNA: luciferase	HEK293 T; Cos7 cells	Able to be modified	Able to be modified	[100]

Abbreviations: DOTAP: 1,2-dioleoyl-3trimethylammonium-propane; DOPE: dioleoylphosphatidyl ethanolamine; DC-cholesterol: 3β-[N-(dimethylaminoethane)carbamoyl] cholesterol; DOPC: 1,2-Dioleoyl-sn-Glycero-3-Phosphocholine; POCG-PEI: poly(1,8-octanedio-citric acid)-co-polyethylene glycol grafted with polyethyleneimine; PEG: polyethylene glycol; PEI: polyethylenimine; MPC: β-cyclodextrin-cross-linked low molecular PEI conjugated with MC11 peptide (MQLPLATGGGC); Ad-SS-PEG: PEG and adamantyl group linked by a disulfide bond; mPEG-PCL: methoxypoly(ethylene glycol)–poly(caprolactone); EGF: epidermal growth factor; VLP: virus-like particle; JCPyV: neurotropic JC polyomavirus; GFP: green fluorescence protein; YFP: yellow fluorescence protein; HSV-TK: herpes simplex virus thymidine kinase type 1 gene; ARCA: anti-reverse cap analogue; Cyc-B1: cyclin B1; CHO cells: Chinese hamster ovary cell line; SKOV-3 cells: ovarian cancer cell line; PC3 cells: human prostate cancer cell line; U87 cells: human primary glioblastoma cell line; DC 2.4 cells: mouse dendritic cells; HuH7 cells: hepatocyte-derived carcinoma cell line; THP-1: human monocytic cell line; iPS cells: induced pluripotent stem cells; U-87 MG: uppsala 87 malignant glioma; DLBCL: diffuse large B-cell lymphoma; ABC-like DLBCL: activated B-cell-like diffuse large B-cell lymphoma; MCF-7: Michigan Cancer Foundation-7 (breast cancer cell); COS-7 cells: monkey kidney fibroblast-like cell; HEK293 cells: human embryonic kidney 293 cells; BSC-40: continuous line of African green monkey cells derived from BSC-1 cells (kidney cells); NIH 3T3 cells: mouse embryonic fibroblast cells; A549 cells: adenocarcinomic human alveolar basal epithelial cells. COLO-320 HSR: Human colon carcinoma cells.
